# Parathyroid Autofluorescence—How Does It Affect Parathyroid and Thyroid Surgery? A 5 Year Experience

**DOI:** 10.3390/molecules24142560

**Published:** 2019-07-14

**Authors:** Roland Ladurner, Maximilian Lerchenberger, Norah Al Arabi, Julia K. S. Gallwas, Herbert Stepp, Klaus K. J. Hallfeldt

**Affiliations:** 1 Department of Surgery, Ludwig Maximilians University Munich, Innenstadt Medical Campus, Nussbaumstrasse 20, 80336 Munich, Germany; 2 Department of Obstetrics and Gynecology, Ludwig Maximilians University Munich, Maistr. 11, 80337 Munich, Germany; 3 Laser-Research Laboratory, LIFE-Center, Ludwig Maximilians University Munich, Grosshadern Medical Campus, Feodor-Lynen-Str. 19, 81377 Munich, Germany; 4 Department of Urology, Ludwig Maximilians University Munich, Grosshadern Medical Campus, Marchioninistrasse 15, 81377 Munich, Germany

**Keywords:** parathyroid, autofluorescence, hypoparathyroidism, near-infrared light, NIR, ICG

## Abstract

Injury to parathyroid glands during thyroid and parathyroid surgery is common and postoperative hypoparathyroidism represents a serious complication. Parathyroid glands possess a unique autofluorescence in the near-infrared spectrum which could be used for their identification and protection at an early stage of the operation. In the present study parathyroid autofluorescence was visualized intraoperatively using a standard Storz laparoscopic near-infrared/indocyanine green (NIR/ICG) imaging system with minor modifications to the xenon light source (filtered to emit 690 nm to 790 nm light, less than 1% in the red and green above 470 nm and no blue light). During exposure to NIR light parathyroid tissue was expected to show autofluorescence at 820 nm, captured in the blue channel of the camera. Over a period of 5 years, we investigated 205 parathyroid glands from 117 patients. 179 (87.3%) glands were correctly identified by their autofluorescence. Surrounding structures such as thyroid, lymph nodes, muscle, or adipose tissue did not reveal substantial autofluorescence. We conclude that parathyroid glands can be identified by their unique autofluorescence at an early stage of the operation. This may help to preserve these fragile structures and their vascularization and lower the rate of postoperative hypocalcemia.

## 1. Introduction

With a size of only a few millimeters and being embedded in adipose tissue, parathyroid glands are often difficult to find intraoperatively. Their blood supply is delicate and can be impaired easily during the operation. Damage to all four glands results in hypoparathyroidism and hypocalcemia. Permanent hypocalcemia following thyroid surgery is seen in 2–5% and represents a serious complication that is difficult to treat, leads to life-long morbidity, and severely compromises the quality of life [1,2,3,4]. Therefore, it would be desirable to find a simple and reliable technique to identify parathyroid glands intraoperatively.

Over the last decades, various intraoperative localization techniques have been proposed to facilitate parathyroid identification. One of the first attempts has been the intravenous injection of methylene blue as a staining agent as it is taken up easily by endocrine tissue [5,6]. Although this method was regarded to be very sensitive, the doses of up to 7.5 mg/kg used at that time showed adverse effects, especially neurotoxic reactions in conjunction with serotonin reuptake inhibitors [7,8,9,10,11]. In recent years, methylene blue has been administered at much lower doses of 0.4–0.5 mg/kg to identify parathyroid glands by fluorescence [6,12,13].

5-aminolevulinic acid (5-ALA) is a metabolic targeting agent and the precursor of the fluorophore protoporphyrin (PpIX) in the heme synthesis pathway [14,15,16]. It is hypothesized that the high number of mitochondria in parathyroid cells leads to the selective accumulation of PpIX in parathyroid glands. Parathyroid fluorescence can be seen 1–8 hours after oral administration of 20–30 mg 5-ALA/kg. However, a major problem is the need to protect patients from direct light exposure for at least 24 hours in order to avoid phototoxic effects on skin and eyes [14,15,16]. 

Indocyanine green (ICG) is the most common fluorophore used at present. When injected intravenously, ICG directly binds to plasma proteins and circulates in the intravascular space until it is taken up by hepatocytes and excreted through the hepatobiliary system. It exhibits fluorescence in the near-infrared (NIR) spectrum and for several decades has been used in many fields of medicine, mainly for fluorescent angiography [17,18,19]. The intensity of the fluorescence is proportional to the blood flow and as parathyroid glands possess an above-average vascularity they are expected to exhibit a stronger fluorescence than the surrounding tissue. In parathyroid surgery, ICG is used to assess parathyroid vascularisation [20,21,22,23,24]. 

10 years ago, a research team of biomedical engineers and endocrine surgeons from Vanderbilt University, Nashville, Tennessee discovered the unique autofluorescence of parathyroid glands [25]. At an excitation wavelength in the near-infrared (NIR) around 800 nm, autofluorescence is emitted at around 820 nm, which is up to 11 times higher than that of the surrounding tissue. As most tissue types are almost completely void of autofluorescence in the near-infrared spectrum, even a weak fluorescence signal can provide a high contrast [26,27,28,29,30,31,32,33,34,35,36,37]. 

Nearly 5 years ago, we introduced intraoperative autofluorescent imaging as a routine investigation to detect parathyroid glands during thyroid and parathyroid surgery. In the following, we will report on our experience applying a commercially available slightly modified NIR/ICG endoscopic imaging system which we also use for standard laparoscopic surgery.

## 2. Results

Between October 2014 and April 2019, 205 parathyroid glands from 117 patients who underwent open thyroid or parathyroid surgery were examined with regard to their autofluorescence. The descriptive data are listed in Table 1.

**Figure 1:**
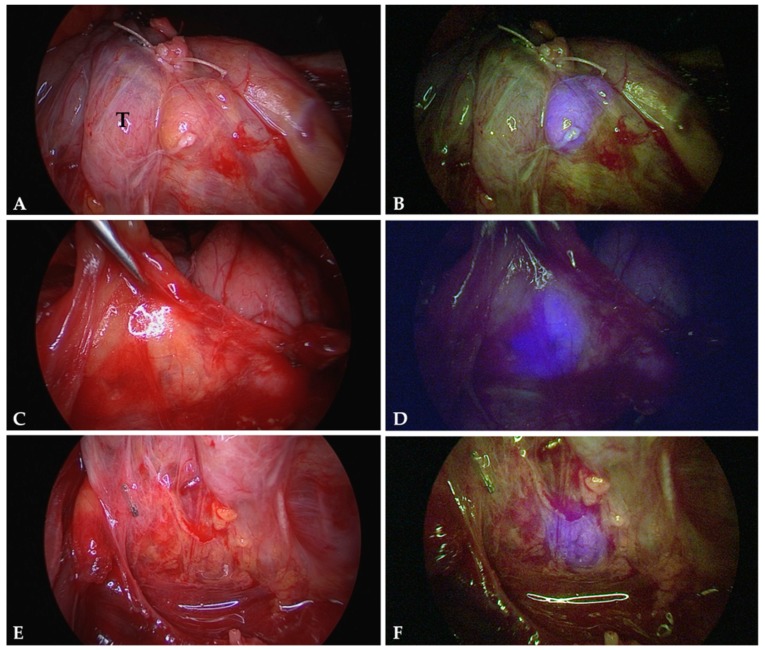
Autofluorescence of normal parathyroid glands. The images correspond to parathyroid glands exposed to normal white light (**A**,**C**,**E**) and near-infrared light (**B**,**D**,**F**). Parathyroid tissue displays a well recognizable bluish violet colour representing autofluorescence. The surrounding structures remain nonfluorescent. Note the different appearances of parathyroid tissue: a parathyroid gland stuck to the thyroid surface (T) (**A**,**B**); a parathyroid gland distant to the thyroid and covered by adipose tissue (**C**,**D**); and parathyroid tissue well hidden in between lymphatic and adipose tissue (**E**,**F**).

**Figure 2:**
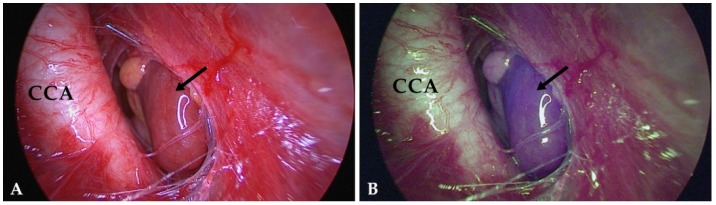
Image of a parathyroid adenoma (arrow) hidden in the vascular sheath close to the common carotid artery (CCA): (**A**) exposed to white light, (**B**) exposed to NIR light. Much like normal parathyroid glands, adenomas display a well recognizable bluish violet color. AF does not allow to distinguish between these two entities.

179 parathyroid glands (87.3%) displayed NIR autofluorescence showing a typical bluish violet color (Figure 1). This fluorescence was sufficient to distinguish parathyroid tissue from surrounding lymph nodes and adipose tissue. Autofluorescence was independent of parathyroid vascularization. Furthermore, we could not discover a difference between normal parathyroid tissue and parathyroid adenomas regarding the intensity of fluorescence (Figure 2). However, we did not carry out quantitative fluorescence measurements.

**Figure 3:**
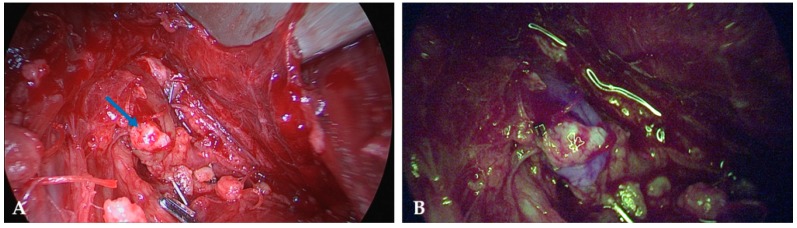
Image of an inferior parathyroid gland identified during central lymph node dissection and stuck to a lymph node (blue arrow) and extremely difficult to recognize when exposed to white light (**A**). In the NIR mode (**B**) the parathyroid tissue displays a well recognizable bluish violet color.

Autofluorescence imaging was especially helpful in identifying inferior parathyroid glands during central lymph node dissection. Often, they are well covered by adipose tissue or hidden in between lymph nodes, and therefore difficult to identify (Figure 3). In most cases their blood supply needs to be sacrificed in consequence of a radical lymphadenectomy. In order to preserve at least part of the parathyroid tissue, these glands need to be autotransplanted which means that the tissue is cut into small pieces and placed in well-vascularized pouches of the sternocleidomastoid muscle. However, it is indispensable to be certain not to transplant lymphatic tissue potentially infiltrated by tumor cells. In this situation, autofluorescence imaging is especially useful to distinguish between parathyroid and lymphatic tissue. 

In 26 cases (12.7%) we were unable to visualize autofluorescence despite definite visual identification of the parathyroid gland. Regarding the extra time necessary to perform AF imaging, we required approximately 3–5 minutes to prepare the imaging system and 2 to 3 min to visualize both parathyroid glands on each side. 

**Figure 4:**
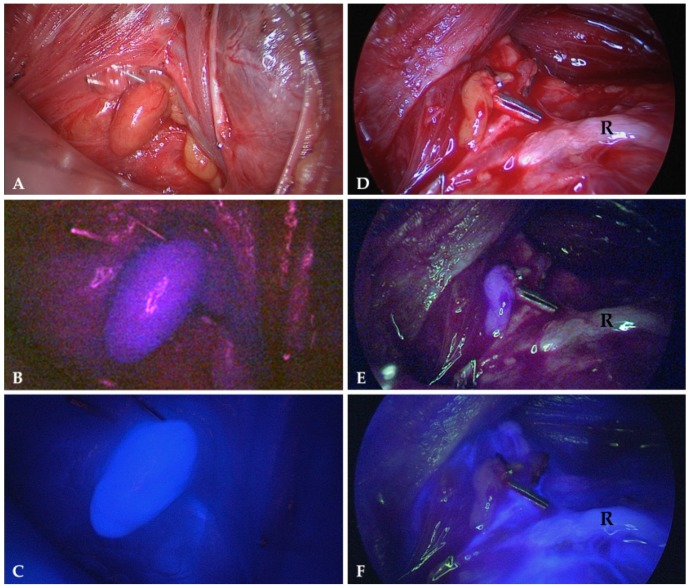
Parathyroid glands exposed to normal white light (**A**,**D**), NIR light displaying their autofluorescence (**B**,**E**) and NIR light 2 min after intravenous application of 5mg indocyanine green (**C**,**F**). The gland on the left displays a strong fluorescence verifying a good vascularity (**C**) while the gland on the right is non-fluorescent (**F**) indicating avascularity (R = recurrent laryngeal nerve).

In selected cases, we verified parathyroid vascularization by indocyanine green (ICG) fluorescent imaging. Two to three minutes after intravenous application of 5mg ICG, parathyroid glands were expected to exhibit a bright fluorescence, far more intense than the surrounding tissue (Figure 4). The absence of fluorescence proved avascularity and justified autotransplantation.

## 3. Discussion

We were able to identify parathyroid glands by their unique autofluorescence with a sensitivity of 87.3%. The technique is especially helpful in complex parathyroid surgery with complete cervical exploration and in thyroid surgery for thyroid cancer with central lymph node dissection. The point was not to screen the operating field for parathyroid glands but to confirm their presence in order to preserve their vascularization. There is a particular high risk of overseeing or injuring the inferior parathyroid glands while performing a central lymph node dissection. Several times, we were able to identify them at an early stage of the procedure and subsequently preserve their vascularization. Even when identified in an avascular state or within the resected specimen the parathyroid tissue was easily distinguished from lymphatic- or adipose tissue and autotransplanted into the sternocleidomastoid muscle without the risk of spreading tumor cells. 

**Figure 5:**
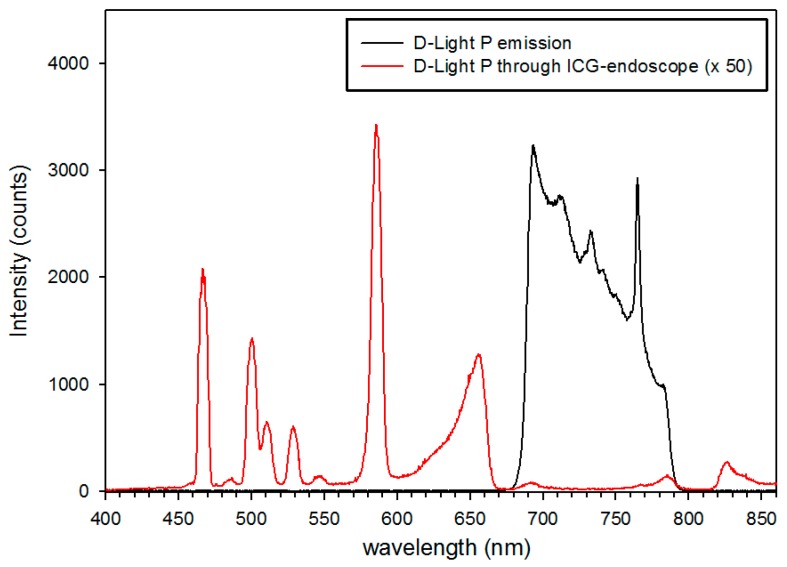
Light spectra emitted from the illumination fibers of the endoscope with the light source in NIR mode (black line) and transmitted through the eyepiece of the endoscope with the endoscope directed to a non-fluorescent reflectance standard (red line). Note: the emission at 465 nm was later removed by a longpass filter.

The sensitivity of 87.3% in our series is slightly low compared to other studies reporting a sensitivity of well above 90% [28,29,30,34,38]. However, it needs to be mentioned that our imaging system had been developed to visualize ICG fluorescence but not the considerably weaker parathyroid autofluorescence. Even after additional filtering, there was still some spurious blue light and NIR light illuminating the tissue. Specular reflections of such light are still visible in the NIR autofluorescence images and diffuse backscatter of such light constitutes an unspecific background. In order to remove these residual artifacts, the light source would have to be re-designed, as one part of the problem arises from scattered light within the light source housing. The other part of the problem is a small peak at 830 nm (Figure 5), which is generated within the illuminating light guides. It can only be removed by a shortpass filter mounted at the distal end of the endoscope’s illumination fibers. However, such a device is not feasible intraoperatively.

**Figure 6:**
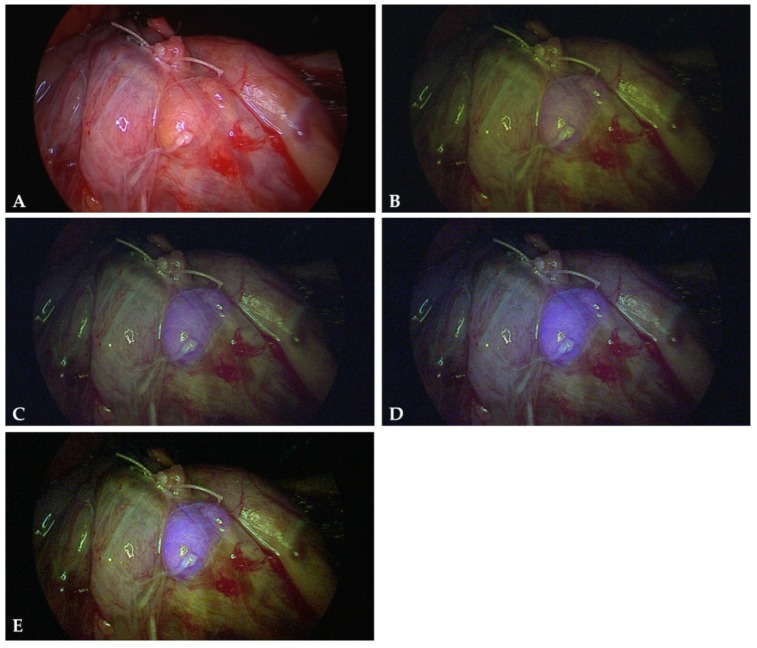
Contrast improvement by simple image processing: original white light (**A**) and NIR-autofluorescence image of a normal parathyroid gland (**B**). (**C**,**D**): full image brightness and contrast adjustments for the blue channel yield a much clearer discrimination of the parathyroid. An additional nonlinear gain setting for the blue channel (gamma set to 1.5) further improves the contrast (**E**). In this example, the contrast of blue intensity in the parathyroid versus green intensity in adjacent tissue could be increased from 0.85 (**B**) to 1.63 (**E**).

**Figure 7:**
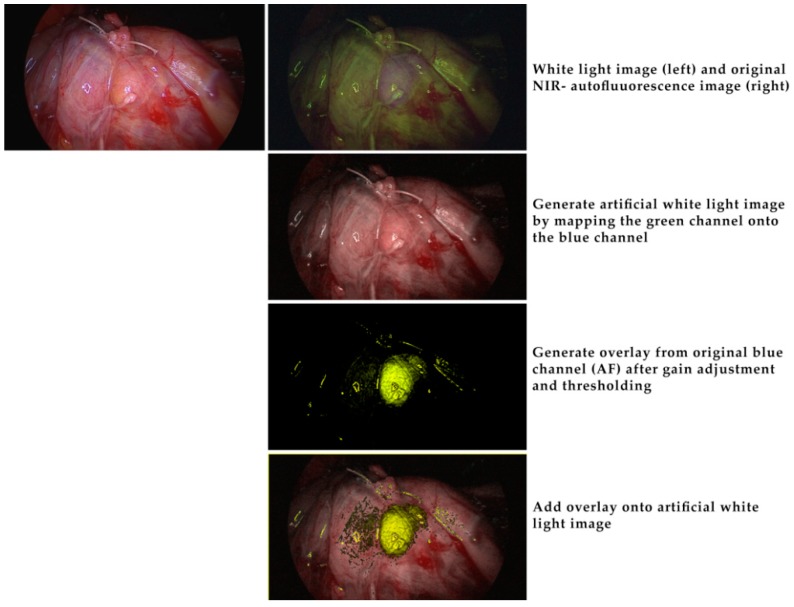
Offline image processing to demonstrate the potential of a future implementation of simple image processing algorithms in a real-time endoscopic NIR-autofluorescence imaging system. Top row: white light image (left) and original NIR-autofluorescence image (right) without any processing. In this image the blue channel was first replaced with the green channel and the gain of the red and green channels slightly adjusted to yield the “artificial” white light image in row 2. The original blue channel, representing the NIR, was processed as described in Figure 6 then thresholded and given a yellowish-green color (row 3). This was then overlaid to the “artificial” white light image. All processing can be implemented in a real time image processing unit.

Due to these limitations, the Storz^®^ system cannot be applied as a real screening tool, identifying parathyroid tissue directly in the initial stage of the operation. Especially when parathyroid glands are covered with adipose tissue, autofluorescence imaging does not replace meticulous dissection by an experienced endocrine surgeon. An approach to improve the current system could be applying a stronger and spectrally improved light source and a more sensitive camera. With some minor offline image enhancement and processing, the potential of the endoscopic system could already be demonstrated (Figure 6). A considerably higher gain setting of the camera’s blue channel during NIR-autofluorescence imaging is certainly feasible, although at the cost of some noise. Also, a re-design of the light source’s light path should further improve the contrast in the NIR-autofluorescence images. Realtime pseudo-colour overlay images on “artificial” white light images, such as depicted in Figure 7 should easily be achievable. In this case, NIR-autofluorescence imaging would easily be implemented during (para)thyroid surgery as a valuable adjunct to rapidly and reliably localize the glands and after surgery check their perfusion by ICG injection (Figure 4). 

Table 2 summarizes the clinical studies so far investigating autofluorescence imaging. A group of biomedical engineers from Vanderbilt University, Nashville, Tennessee was the first to describe autofluorescence in 2011 [25]. So far they have investigated 264 patients spectroscopically and reported detection rates between 97% and 100% [28]. However, spectroscopic measurements require direct contact with the tissue which is impractical in a surgical setting. In contrast, imaging cameras not only work contactless but also provide a large field of view which constitutes a great advantage intraoperatively. Several studies were carried out using the commercially available Fluobeam^®^ 800 system (Fluoptics, Grenoble, France). A laser provides an irradiance of 5 mW/cm^2^ at 750 nm and collects the optical signal for wavelengths above 800 nm [29,30,34,36]. At an experimental level, Kim et al. introduced a new technique to visualize both parathyroid glands and the surrounding tissue in a single image, using a 780 nm collimated light-emitting diode (Thorlabs, Newton, NJ, USA) with an excitation filter appropriate for parathyroid autofluorescence, an illuminator (INFRALUX-300, Daekyoung, Korea) for reflection of the entire surgical area, and a digital single-lens reflex camera (Canon, EOS REBEL T3, Ota, Tokyo, Japan) [33,37]. 

Although it is not perfect and needs further improvements, we decided to hold on to the ICG/NIR Storz^®^ system because of its versatility. Costs represent a major factor in healthcare, and compared to the other techniques discussed this device can also be used for standard laparoscopic surgery, therefore reducing the additional costs to a minimum. 

Clinical reports on NIR application to identify parathyroid glands are continuously increasing, while only little attention has been given to the biochemistry of parathyroid tissue to identify the fluorophores responsible for the high NIR autofluorescence. Currently, most of their characteristics remain unknown. Our present knowledge is mainly based on the work of McWade et al. which we will briefly describe in the following [27,39]. Initial studies characterized the fluorophores as highly robust. Ex vivo fluorescence measurements on bulk tissue showed a remarkable stability with a half-life of 152 hours. Proteinase K, a broad-spectrum protease, led to a strong reduction of NIR fluorescence between 20 and 50 hours of incubation of a parathyroid lysate. The relative resistance to the proteinase exposure for the first 20 hours is certainly unusually high, still indicates that the fluorophore could be a peptide, protein or requires a protein to fluoresce. On the other hand, there was no change of fluorescence in response to heat even at the temperature of boiling water [39,40]. One primary fluorophore candidate has been the extracellular calcium-sensing receptor, which shows highest concentrations in the parathyroid tissue, lower concentrations in the thyroid and is not present in other neck tissues. To support this hypothesis, McWade et al. measured NIR fluorescence in bulk human kidney and colon, which also highly express the calcium-sensing receptor [27,39]. They could demonstrate that these tissues possessed the same peak fluorescence at 822 nm as parathyroid or thyroid tissue when excited with 785 nm. NIR fluorescence microscopy of parathyroid parenchyma cells showed a predominant fluorescence emission originating from the cytoplasma and only little emisssion from the cell nucleus or the plasma membrane. Both main cell types of the parathyroid gland, chief, and oxyphil cells, emitted fluorescence, indicating that this phenomenon is not confined to a single cell type [39,41]. Differential centrifugation of parathyroid tissue showed the greatest autofluorescence to be present in the organellar fraction that is rich with mitochondria, lysosomes and secretory granules. Further investigations performed on human parathyroid tissue sections using chromogranin A as a marker for secretory granules showed a partial colocalization between chromogranin A and NIR autofluorescence, which was almost exclusively present in the cytoplasm, with a punctate pattern in the parathyroid parenchyma cells [32,40]. Studies to separate the NIR fluorophore in parathyroid tissue by gel electrophoresis showed a strong fluorescence at 15 kDa. Knowledge of the molecular weight may be helpful to identify the precise fluorescent protein or peptide in future studies [39]. Evidence has been reported on a similarity between the autofluorescence in the parathyroid, thyroid, pancreas and the adrenal medulla, indicating that the fluorophore is the same in each of these endocrine tissues and related to the production and secretion of hormones [39,42,43]. 

In chapter 9.3 of her thesis, Melanie Ann McWade recommends a series of future experiments, which might finally lead to uncover the precise nature of the fluorophore [39]. We are not aware, however, of any progress in this regard. 

It would be highly desirable if further research identifies and confirms the specific fluorophore(s) responsible for parathyroid NIR autofluorescence, along with their possible role in function and regulation of the glands. This could improve the sensitivity of in situ parathyroid detection in the presence of dysfunction and disease, with possible extension to NIR diagnosis procedures to other endocrine glands such as adrenal medulla and pancreas. Regarding the operative treatment of thyroid and parathyroid disorders, NIR autofluorescence imaging clearly has the potential to identify parathyroid glands at an early stage of the operation. It may help to protect their vascularisation and to minimize postoperative hypocalcemia. 

## 4. Materials and Methods 

### 4.1. Patients

Patients presenting with thyroid disease undergoing partial or total thyroidectomy or presenting with primary hyperparathyroidism undergoing open parathyroidectomy were recruited for this study. Informed consent was obtained from all participants. The study was approved by the Ethical Review Board of the Medical Faculty of the University of Munich (ethical approval codes: 229-14, 17-119). 

### 4.2. Imaging System

Autofluorescence imaging of parathyroid and other tissues was performed with a commercially available near-infrared/indocyanine green (NIR/ICG) endoscopic system (Karl Storz^®^, Tuttlingen, Germany) with adapted excitation filters. The system comprises a xenon light source (D-Light P, Karl Storz), which provides both visible and NIR excitation (690 nm to 790 nm) light, changeable by a foot switch. A high-end full high definition camera system (Image1 H3-Z 3-Chip Full HD camera, Karl Storz) was connected to a 10 mm diameter, 0° ICG endoscope (Hopkins™ II, 26003ACA, Karl Storz^®^). The 3-chip camera is sensitive for NIR light with its blue channel, due to the spectral properties of the dielectric coatings of the color beamsplitter [44]. The endoscope is equipped with a specific filter for optimal transmission of white light and NIR fluorescence, while completely blocking out NIR excitation light. When switched to the NIR excitation mode, the light source also emits low intensity light in the green and red spectral ranges to enable orientation during NIR fluorescence imaging (Figure 5). The peak at 465 nm was eliminated by mounting an additional long pass filter in the light source’s filter wheel. In this configuration, NIR fluorescence is detected by the camera’s blue channel. As there is no blue light emitted from the light source, when switched to NIR mode, any blue signal detected and displayed is indicative of NIR light impinging upon the camera. A kind of simultaneous white light imaging is provided with the green and red channels of the camera. Images were captured with a digital storage device (AIDA compact NEO advanced FULL-HD documentation system, Karl Storz^®^) and processed using ImageJ (National Institutes of Health, Bethesda, MD, USA). Only full image adjustments of contrast and brightness of the separate color channels were made, if not otherwise noted in the figure captions. 

### 4.3. Intraoperative Autofluorescence Imaging 

Autofluorescence (AF) imaging was carried out during parathyroid and thyroid surgery for benign and malignant disease. Special care was taken to visualize the parathyroid glands and to preserve their vascularization. However, we did not search for them when they were not apparent during initial thyroid mobilisation. Images were collected only after definite identification of a parathyroid gland. In most cases, the thyroid had been mobilized laterally but was still in situ. The tip of the laparoscope was held stationary approximately 5 cm above the tissue. First, white light images were collected. Second, with all operating room lights switched off, the parathyroid gland as well as the surrounding tissue were exposed to near-infrared (NIR) light. The parathyroid gland was expected to be displayed in the blue color channel. In the NIR-mode, the light source emitted little green and red light to provide visualization of the surrounding tissue. Therefore, the bluish violet colored parathyroid glands could be seen in their exact anatomical position (Figure 1 and Figure 2). 

### 4.4. Intraoperative ICG Imaging

For ICG fluorescence imaging, 25 mg of the fluorophore ICG-Pulsion^®^ (Diagnostic Green GmbH, Aschheim-Dornach, Germany) were dissolved in 5 mL sterile water and 5 mg (1 mL) injected intravenously. ICG fluorescence became apparent after 1–2 minutes. To allow direct comparison between autofluorescence and ICG fluorescence, we investigated only one side in each patient. Measuring autofluorescence after application of ICG on the contralateral side would have falsified the results of parathyroid AF imaging. 

## Figures and Tables

**Table 1:** Descriptive data.

Number of Patients	117
Mean age	49.9 (19–81) years
Gender (female/male)	76/41
Type of operation	(n)
Open parathyroidectomies	42
Thyroidectomies	75
Goiter	36
Grave’s disease	14
Carcinoma	25

**Table 2:** Clinical studies applying NIR autofluorescence to identify parathyroid glands. (PG = parathyroid gland, P/T = parathyroidectomy/thyroidectomy; T = thyroidectomy).

Authors	Study Design	Surgery	(n)	Device	Detected PG (%)
Paras 2011 [25]	Case series	P/T	21	785 nm diode laser/spectrometer	100%
Mac Wade 2013 [26]	Case series	P/T	45	785 nm diode laser/spectrometer	100%
Mac Wade 2014 [27]	Case series	P/T	110	785 nm diode laser/spectrometer	100%
Mac Wade 2016 [28]	Case series	P/T	264	785 nm diode laser/spectrometer	100%
De Leeuw 2016 [29]	Case series	P/T	35	Fluobeam^®^ 800 clinical system	98.8%
Falco 2016 [30]	Case series	P/T	28	Fluobeam^®^ 800 clinical system	100%
Kim 2016 [33]	Case series	T	8	780 nm LED/single lens reflex camera	100%
Falco 2017 [35]	Multi center series	P/T	74	750 nm laser/imaging system	100%
Ladurner 2017 [31]	Case series	P/T	30	Storz^®^ ICG/NIR imaging system	80.9%
Kahramangil 2018 [36]	Multi center series	P/T	210	Fluobeam^®^ 800 clinical system	98%
Kim 2018 [37]	Case series	T	38	780 nm LED/single lens reflex camera	98.5%
Ladurner 2018 [32]	Case series	T	20	Storz^®^ ICG/NIR imaging system	90.2%
Benmiloud 2018 [34]	Controlled study	T	93	Fluobeam^®^ 800 clinical system	76.3%
